# Supplementary material for: A symbiotic bacterium of shipworms produces a compound with broad spectrum anti-apicomplexan activity
Source: PLoS Pathog. 2020 May 26;16(5):e1008600. doi: 10.1371/journal.ppat.1008600 (PMC7274485; doi:10.1371/journal.ppat.1008600)
Supplement: S1 Fig — HFF cells were infected with T. gondii parasites for 24 hours then medium containing bacterial culture supernatants at final dilutions of 1:100 or 1:250 were added to the infected cells. 24 hours post-treatment cells were fixed and processed for IFAs. Parasites were labeled with rabbit anti-SAG1 antibody detected with Alexafluor 594-labelled goat anti-rabbit IgG (green). Host cell nuclei are visualized with DAPI. Scale bar = 10 μm. (DOCX) [file ppat.1008600.s001.docx]

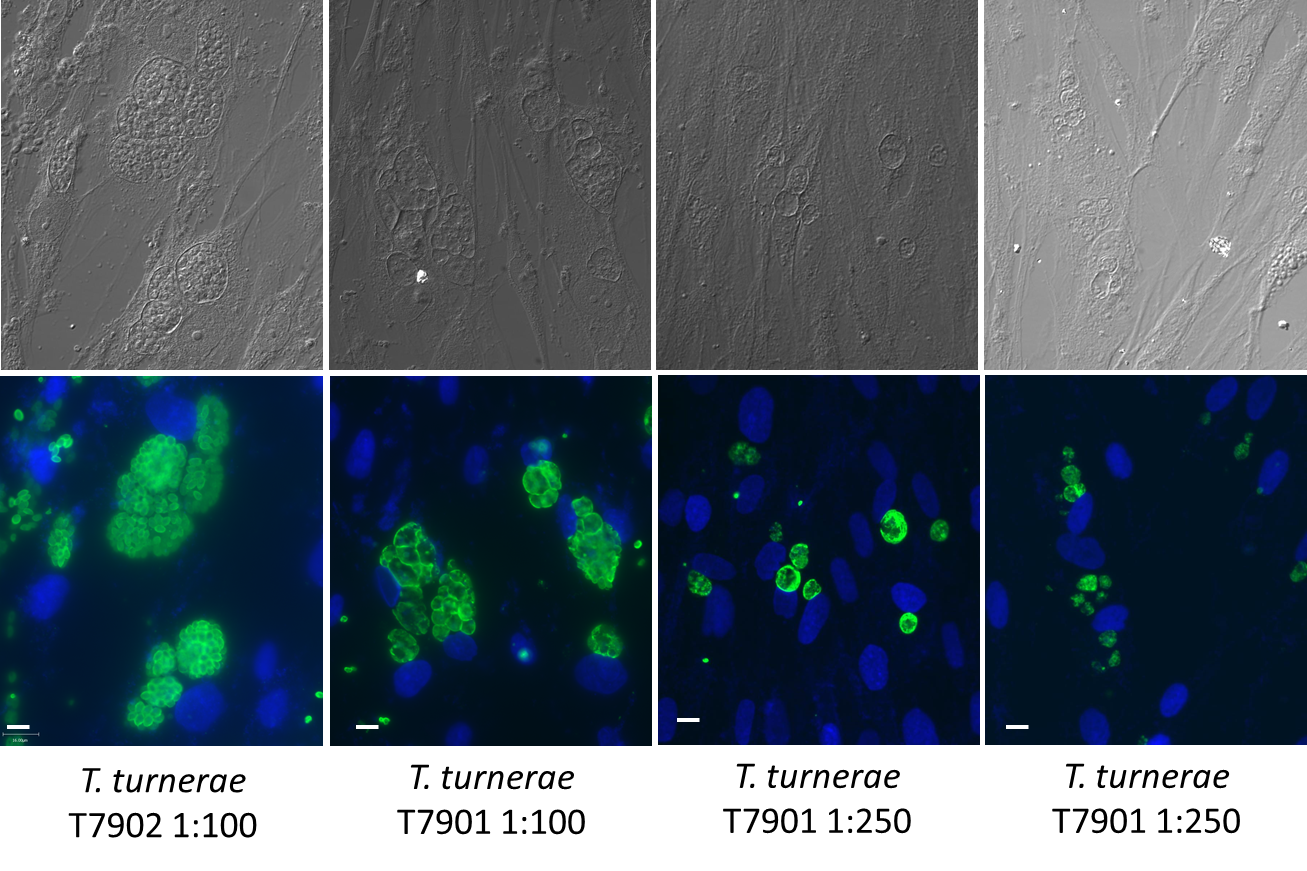


**S1 Fig:** ***T. gondii* treated with culture supernatants from shipworm symbionts T7902 and T7901.**  HFF cells were infected with *T. gondii* parasites for 24 hours then medium containing bacterial culture supernatants at final dilutions of 1:100 or 1:250 were added to the infected cells. 24 hours post-treatment cells were fixed and processed for IFAs. Parasites were labeled with rabbit anti-SAG1 antibody detected with Alexafluor 594-labelled goat anti-rabbit IgG (green). Host cell nuclei are visualized with DAPI. Scale bar=10µm.
