# Supplementary material for: A symbiotic bacterium of shipworms produces a compound with broad spectrum anti-apicomplexan activity
Source: PLoS Pathog. 2020 May 26;16(5):e1008600. doi: 10.1371/journal.ppat.1008600 (PMC7274485; doi:10.1371/journal.ppat.1008600)
Supplement: S2 Fig — Bioassay-guided fractionation of T. turnerae T7901 culture supernatant: A. Methanol/water fractions eluted off a C18 column were lyophilized, resuspended in DMSO and tested for anti-Toxoplasma activity using a monolayer protection assay. Details of the extractions can be found in methods. For the monolayer protection assay, HFF cells were seeded into 96 well plates, infected for 24 hours with T. gondii RH strain parasites, and then dilutions of extracts were added to the infected cells. 24 hours post-treatment the cell monolayers were fixed and stained with trypan blue. B. HFF cells infected with T. gondii RH strain parasites for 24 hours were treated with the 90% methanol fraction diluted to 10 μg/ml or DMSO control. 24 hours post treatment, infected cells were fixed and processed for IFAs. Parasites were labeled with rabbit anti-SAG1 antibody detected with Alexafluor 594-labelled goat anti-rabbit IgG (red). Host cell nuclei are visualized with DAPI. (DOCX) [file ppat.1008600.s002.docx]

**
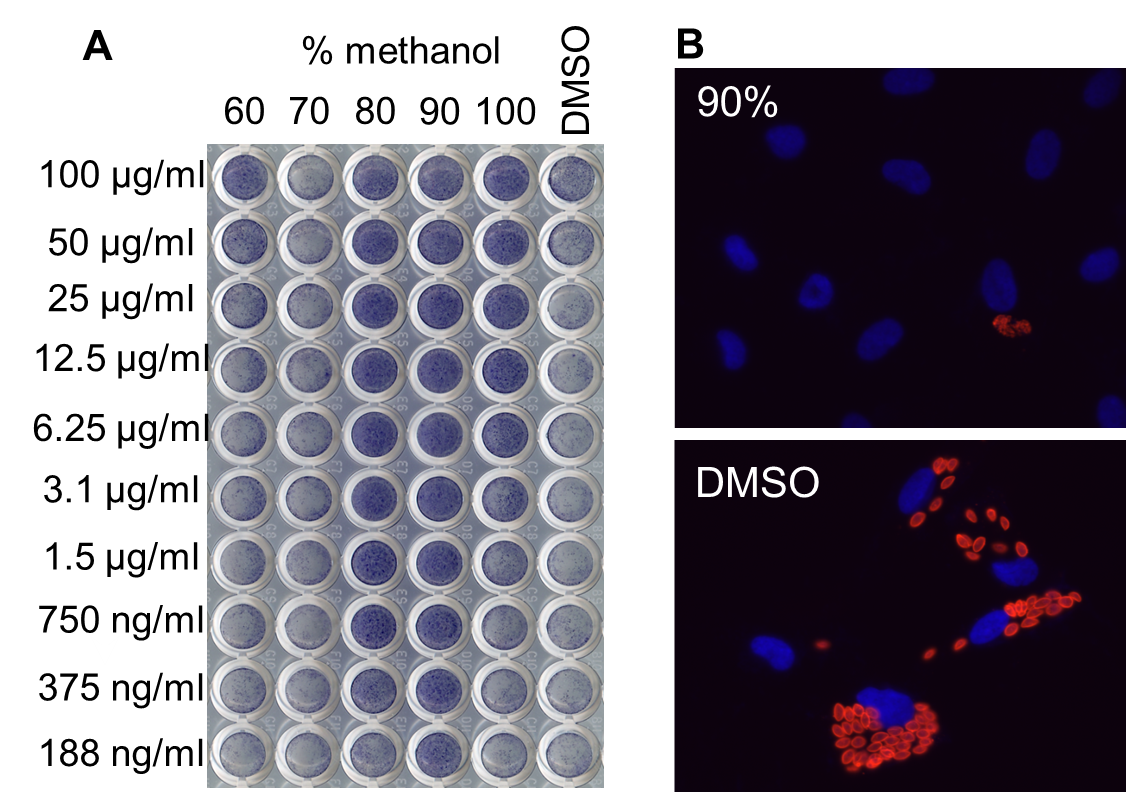
**

**S2 Fig: Bioassay-guided fractionation of *T. turnerae* T7901 culture supernatant**: **A.** Methanol/water fractions eluted off a C18 column were lyophilized, resuspended in DMSO and tested for anti-*Toxoplasma* activity using a monolayer protection assay. Details of the extractions can be found in methods. For the monolayer protection assay, HFF cells were seeded into 96 well plates, infected for 24 hours with *T. gondii* RH strain parasites, and then dilutions of extracts were added to the infected cells. 24 hours post-treatment the cell monolayers were fixed and stained with trypan blue. **B.** HFF cells infected with *T. gondii* RH strain parasites were treated with the 90% methanol fraction diluted to 10 µg/ml or DMSO control. 24 hours post treatment, infected cells were fixed and processed for IFAs. Parasites were labeled with rabbit anti-SAG1 antibody detected with Alexafluor 594-labelled goat anti-rabbit IgG (red). Host cell nuclei are visualized with DAPI.
