# Supplementary material for: A symbiotic bacterium of shipworms produces a compound with broad spectrum anti-apicomplexan activity
Source: PLoS Pathog. 2020 May 26;16(5):e1008600. doi: 10.1371/journal.ppat.1008600 (PMC7274485; doi:10.1371/journal.ppat.1008600)
Supplement: S3 Fig — The purity of trtE was calculated > 99%. (DOCX) [file ppat.1008600.s003.docx]

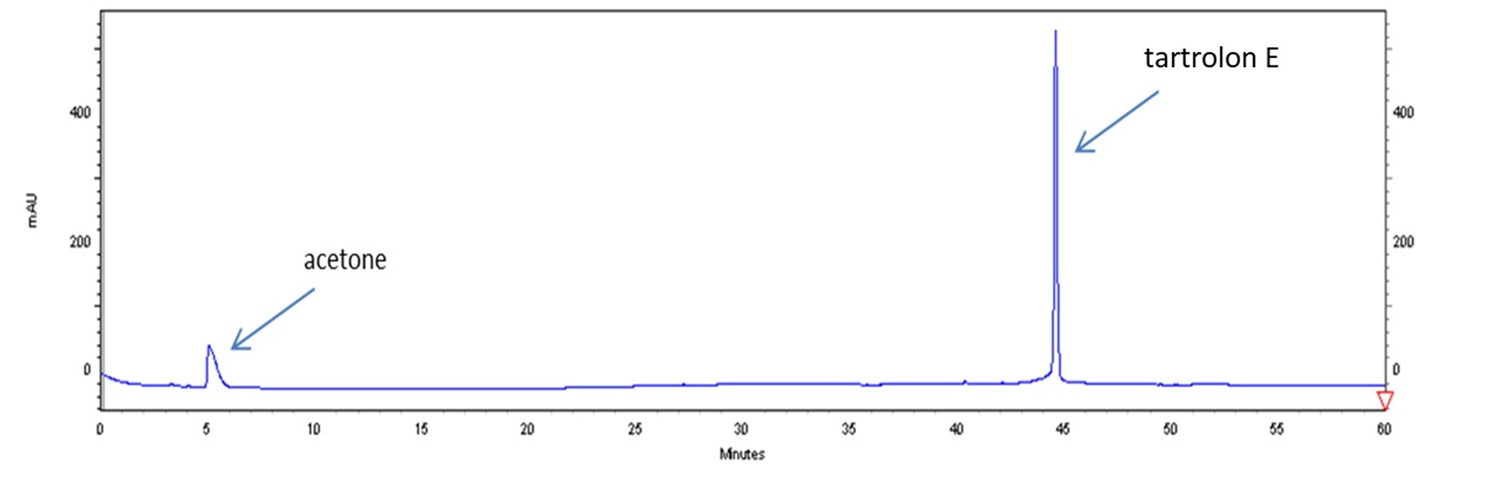


**S3 Fig: HPLC chromatogram of trtE purified by method 1, detected in 224 nm by DAD.** The purity of trtE was calculated > 99%.
