# Supplementary figures and images for: A symbiotic bacterium of shipworms produces a compound with broad spectrum anti-apicomplexan activity
Source: PLoS Pathog. 2020 May 26;16(5):e1008600. doi: 10.1371/journal.ppat.1008600 (PMC7274485; doi:10.1371/journal.ppat.1008600)

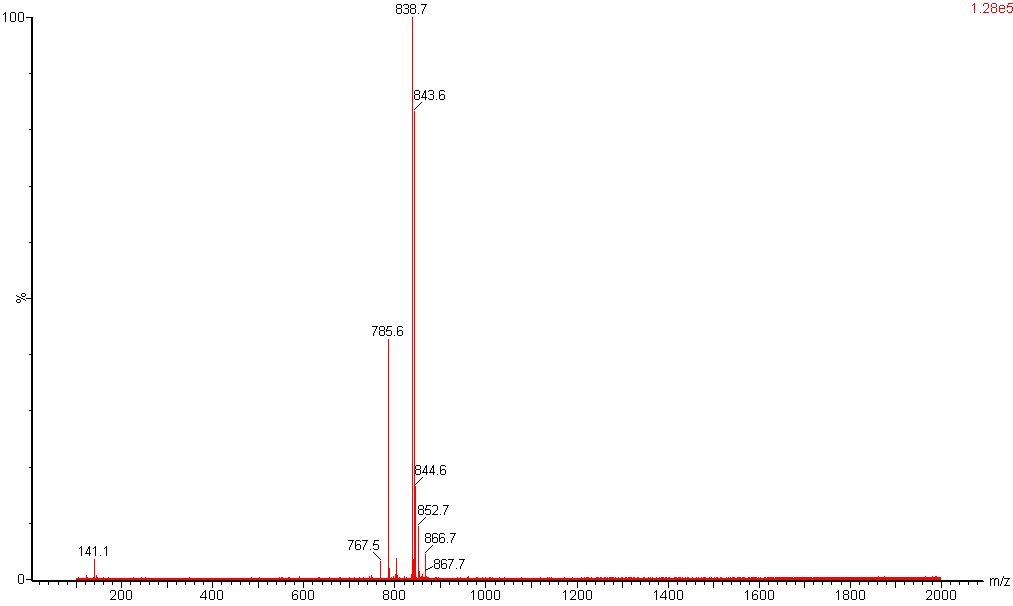


**S4 Fig: Mass spectrometry data of trtE purified by method 1.**

Supplement: S4 Fig — (DOCX) [file ppat.1008600.s004.docx]

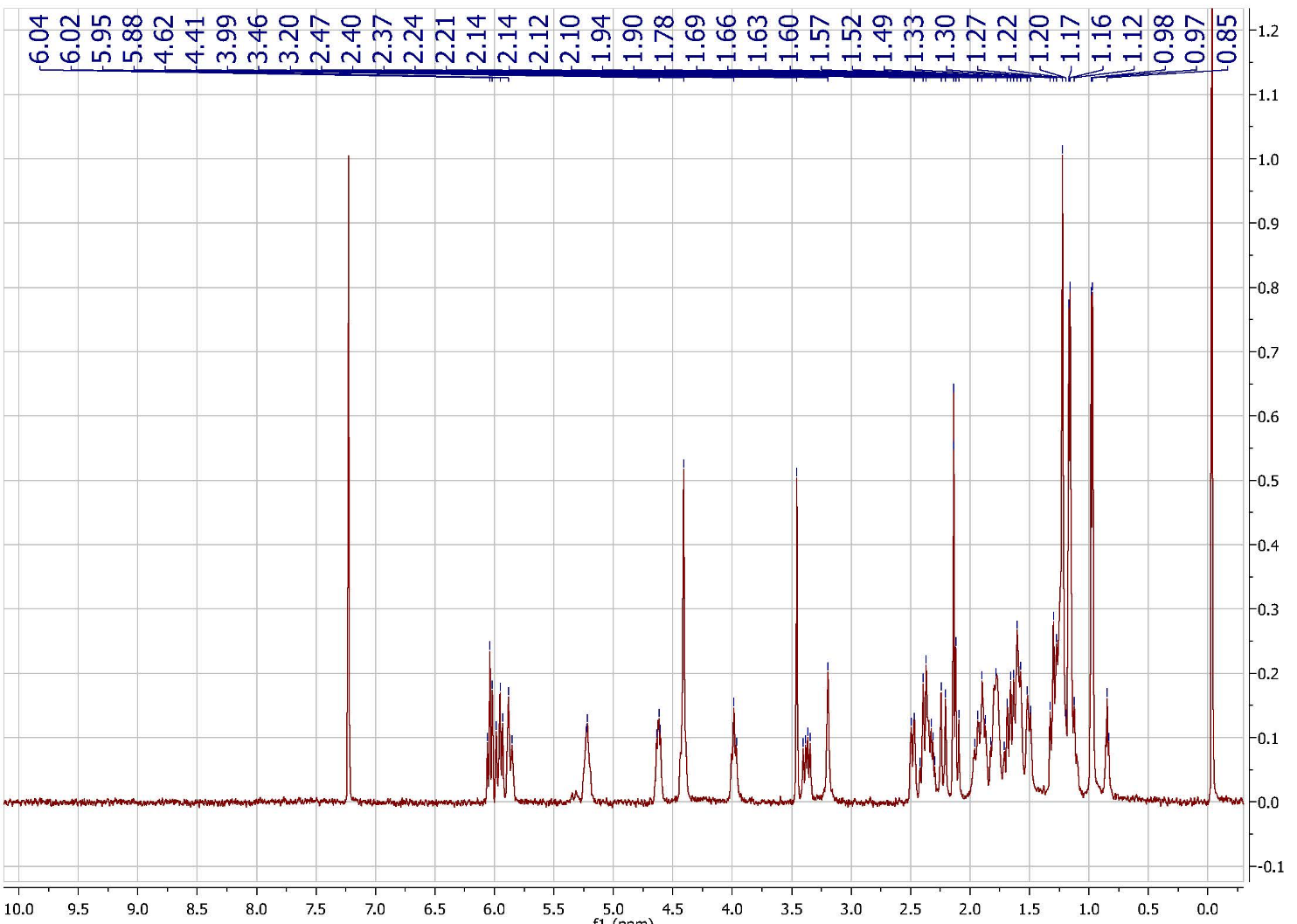


**S5 Fig: 1H NMR spectra in CDCl3 of trtE purified by Method 1.**

Supplement: S5 Fig — (DOCX) [file ppat.1008600.s005.docx]

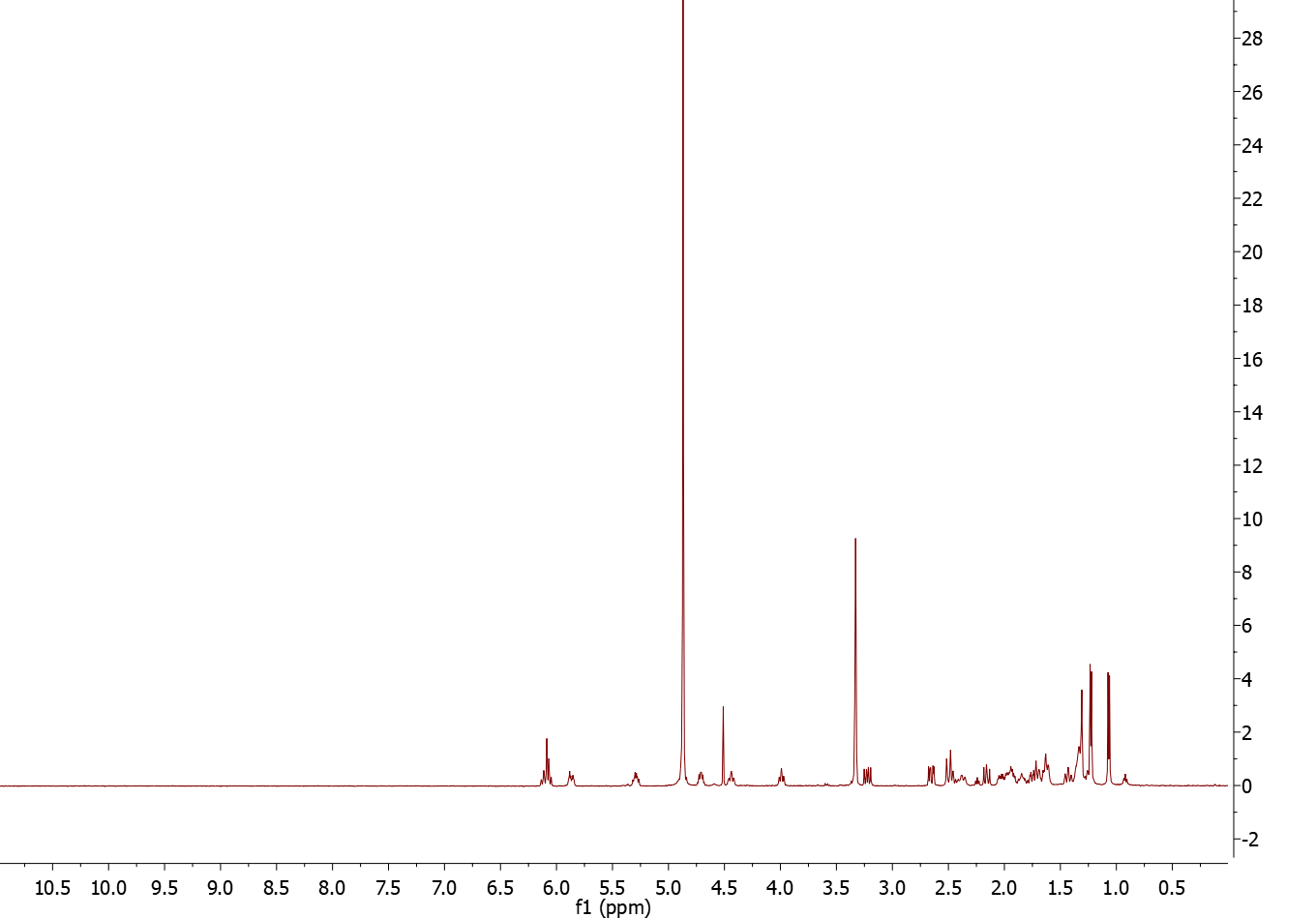


**S6 Fig: 1H NMR spectra in CD3OD of trtE purified by Method 1.**

Supplement: S6 Fig — (DOCX) [file ppat.1008600.s006.docx]

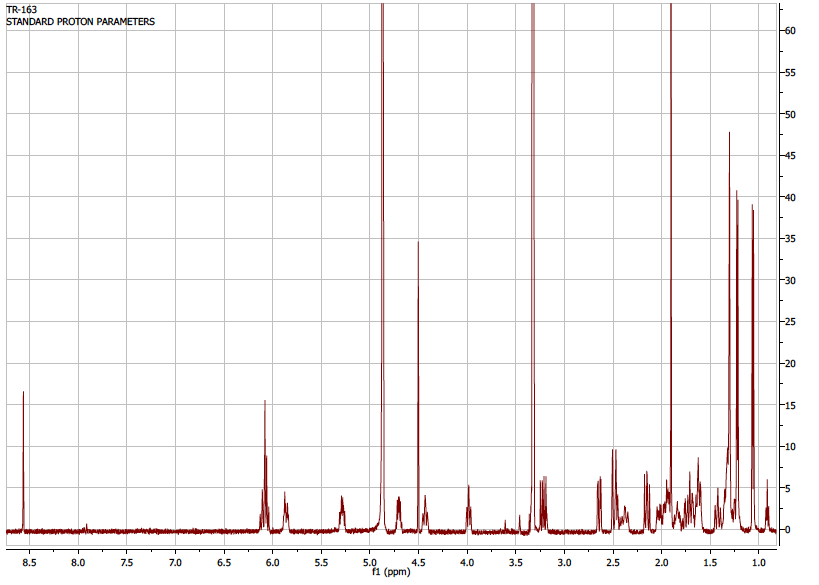


**S9 Fig: 1H NMR spectra of trtE purified by Method 2 (500 MHz, CD3OD).**

Supplement: S9 Fig — (DOCX) [file ppat.1008600.s009.docx]
