# Supplementary material for: A symbiotic bacterium of shipworms produces a compound with broad spectrum anti-apicomplexan activity
Source: PLoS Pathog. 2020 May 26;16(5):e1008600. doi: 10.1371/journal.ppat.1008600 (PMC7274485; doi:10.1371/journal.ppat.1008600)
Supplement: S7 Fig — T. gondii RH strain tachyzoites were allowed to infect HFF cells for 24 hours at which point trtE was added to a final concentration of 60nM. Cells were fixed and processed for IFA 24 hours after the addition of the compound. DMSO was run in parallel as a negative control. Images on the left are DIC, images on the right show the IFA. Parasites are labeled with rabbit anti-SAG1 antibody detected with Alexafluor594-labeled goat anti-rabbit IgG (red). Host cell nuclei are visualized with DAPI (blue). Panel A shows parasites treated with DMSO. Panels B through F show infected cells treated with trtE. Scale bar = 10μm. (DOCX) [file ppat.1008600.s007.docx]

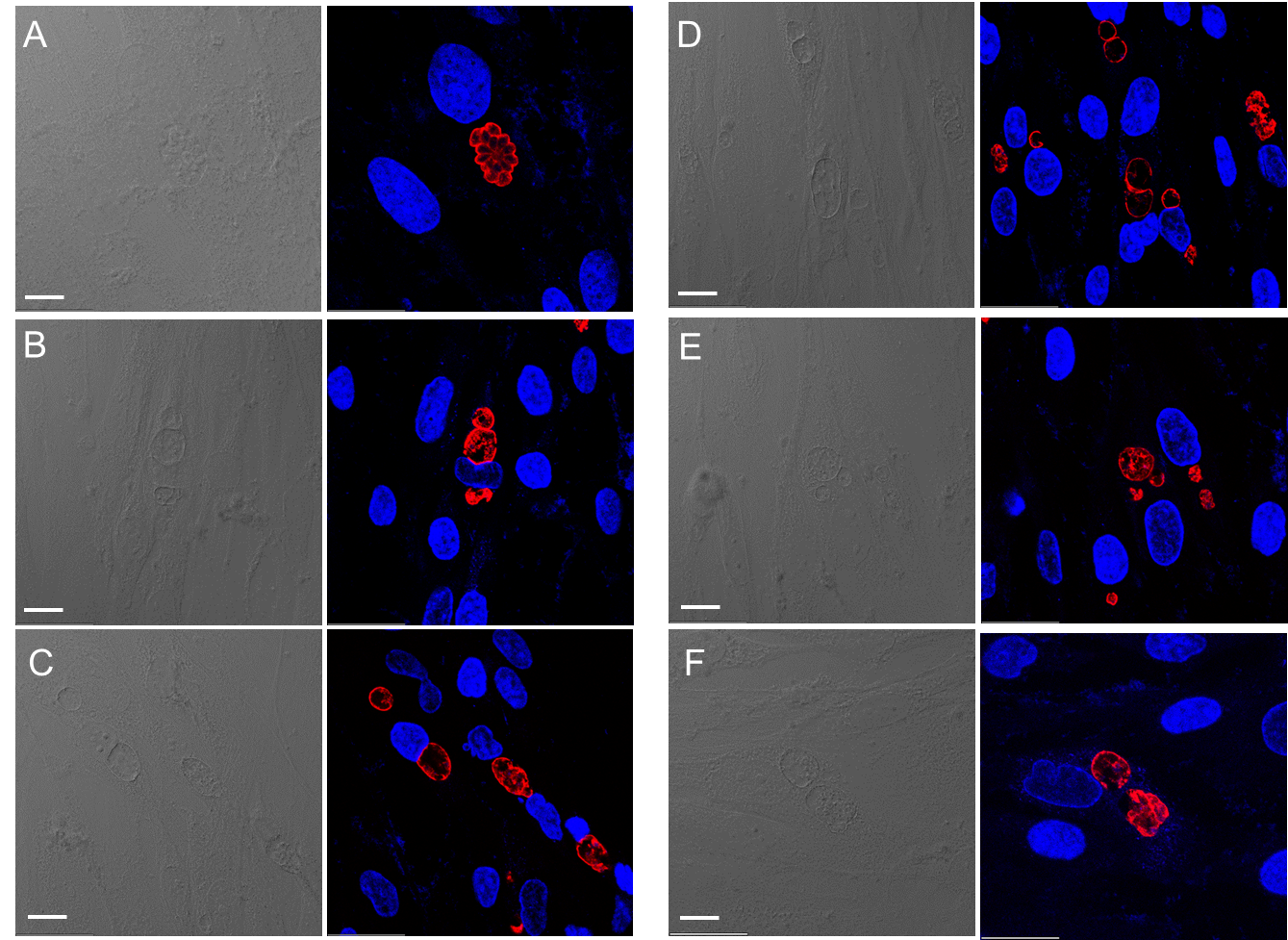


**S7 Fig: Intracellular stages of *Toxoplasma gondii* treated with trtE:** *T. gondii* RH strain tachyzoites were allowed to infect HFF cells for 24 hours at which point trtE was added to a final concentration of 60nM. Cells were fixed and processed for IFA 24 hours after the addition of the compound. DMSO was run in parallel as a negative control. Images on the left are DIC, images on the right show the IFA. Parasites are labeled with rabbit anti-SAG1 antibody detected with Alexafluor594-labeled goat anti-rabbit IgG (red). Host cell nuclei are visualized with DAPI (blue). Panel **A** shows parasites treated with DMSO. Panels **B** through **F** show infected cells treated with trtE. Scale bar =10 µm.
