# Supplementary material for: A symbiotic bacterium of shipworms produces a compound with broad spectrum anti-apicomplexan activity
Source: PLoS Pathog. 2020 May 26;16(5):e1008600. doi: 10.1371/journal.ppat.1008600 (PMC7274485; doi:10.1371/journal.ppat.1008600)
Supplement: S8 Fig — (DOCX) [file ppat.1008600.s008.docx]

**S8 Fig: LC-MS data of trtE purified by Method 2.**
