# Supplementary material for: A symbiotic bacterium of shipworms produces a compound with broad spectrum anti-apicomplexan activity
Source: PLoS Pathog. 2020 May 26;16(5):e1008600. doi: 10.1371/journal.ppat.1008600 (PMC7274485; doi:10.1371/journal.ppat.1008600)
Supplement: S10 Fig — HCT-8 cells were infected with C. parvum oocysts for 8 hours, at which time cells were washed to remove extracellular parasites and medium containing 60 nM trtE or DMSO was added to the infected cells. 12 hours later, infected cells were fixed and processed for IFAs. Images on the left are DIC, images on the right show the IFA. Parasites are labeled with rabbit anti-gp15 antibody detected with Alexafluor594-labeled goat anti-rabbit IgG (red). Host cell nuclei are visualized with DAPI (blue). Panels A through C show parasites treated with DMSO. Panels D through F show parasites treated with trtE. Very few discernable parasites could be found in the trtE treated cells. Scale bar = 5μm. (DOCX) [file ppat.1008600.s010.docx]

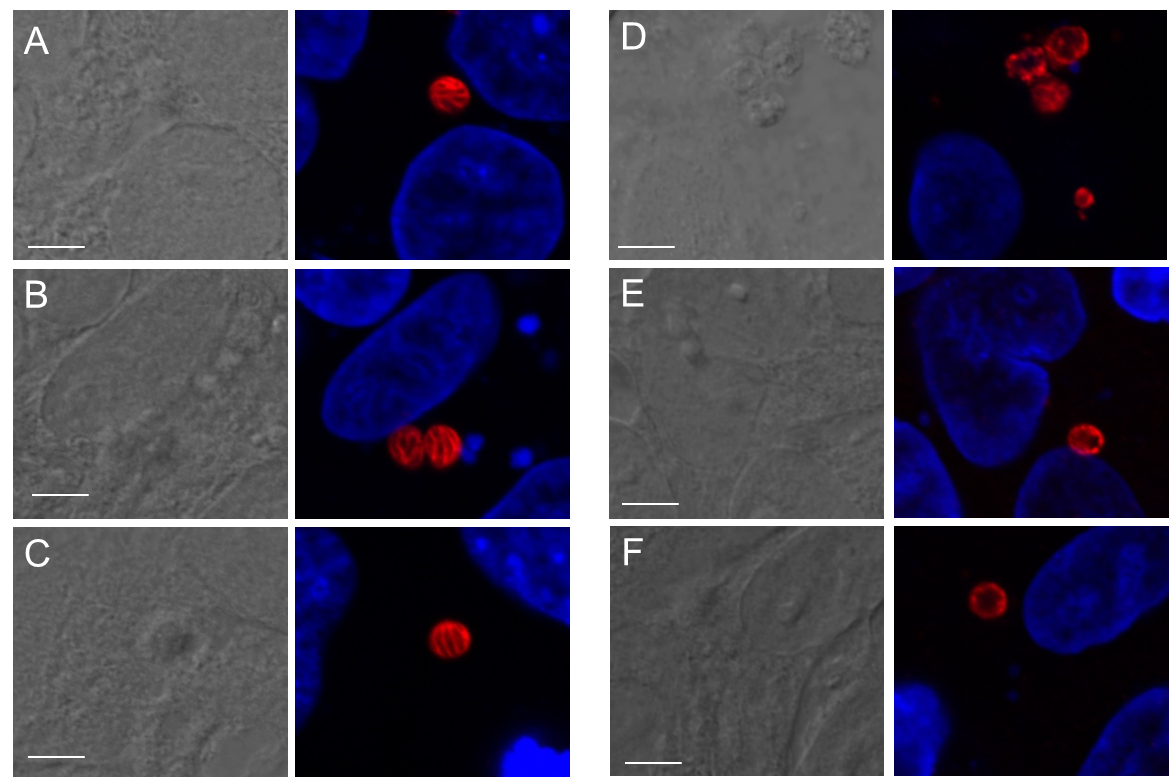


**S10 Fig: Intracellular *C. parvum* parasites treated with trtE:** HCT-8 cells were infected with *C. parvum* oocysts for 8 hours, at which time cells were washed to remove extracellular parasites and medium containing 60 nM trtE or DMSO was added to the infected cells. 12 hours later, infected cells were fixed and processed for IFAs. Images on the left are DIC, images on the right show the IFA. Parasites are labeled with rabbit anti-gp15 antibody detected with Alexafluor594-labeled goat anti-rabbit IgG (red). Host cell nuclei are visualized with DAPI (blue). Panels **A** through **C** show parasites treated with DMSO. Panels **D** through **F** show parasites treated with trtE. Very few discernable parasites could be found in the trtE treated cells. Scale bar = 5 µm.
