# Supplementary material for: A symbiotic bacterium of shipworms produces a compound with broad spectrum anti-apicomplexan activity
Source: PLoS Pathog. 2020 May 26;16(5):e1008600. doi: 10.1371/journal.ppat.1008600 (PMC7274485; doi:10.1371/journal.ppat.1008600)
Supplement: S11 Fig — A. Bovine turbinate cells infected with luciferase expressing Sarcocystis neurona merozoites were treated with trtE for 24 hours and parasite growth evaluated by luciferase expression. EC50s were determined using the log[inhibitor]vs response-Variable slope (four parameter) regression equation in Graphpad Prism, EC50 = 12.9 nM with a 95%CI of 11–15 nM. B. Theileria equi-infected erythrocytes were incubated with trtE or DMSO vehicle control and proliferation was measured after 72 hours by SYBR green incorporation. Percent proliferation was calculated relative to DMSO treated controls. Inhibition of proliferation was analyzed as described for (A), EC50 = 391 pM (95% CI 286–550 pM). (DOCX) [file ppat.1008600.s011.docx]

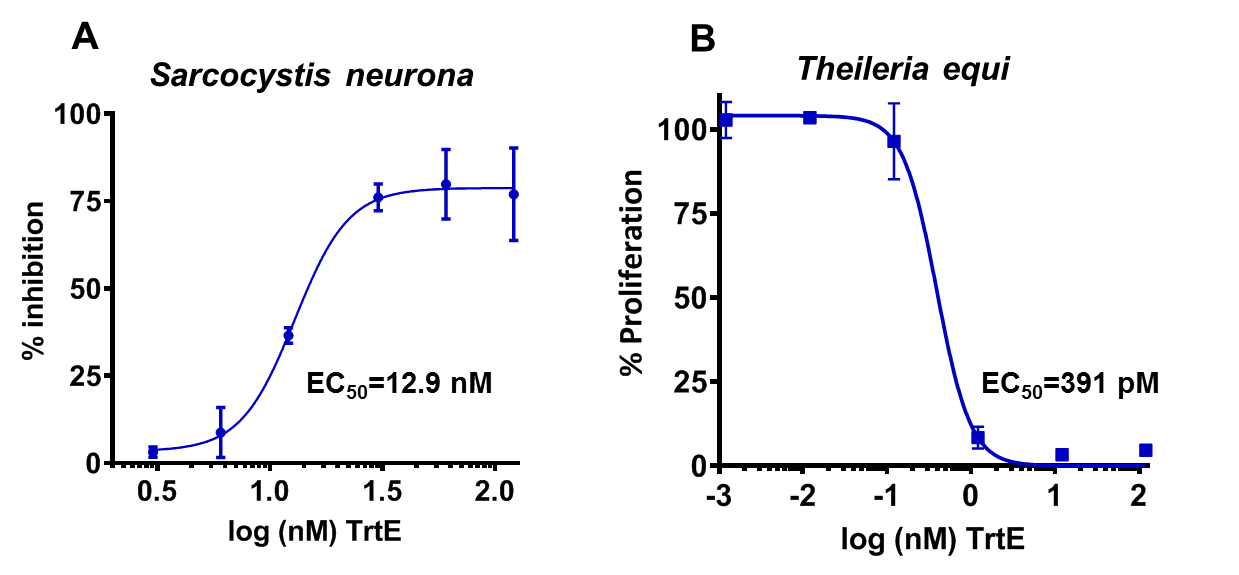


**S11 Fig: TrtE exhibits broad spectrum anti-apicomplexan activity in vitro. A.** Bovine turbinate cells infected with luciferase expressing *Sarcocystis neurona* merozoites were treated with trtE for 24 hours and parasite growth evaluated by luciferase expression. EC_50_s were determined using the log[inhibitor]vs response-Variable slope (four parameter) regression equation in Graphpad Prism, EC_50_=12.9 nM with a 95%CI of 11-15 nM. **B**.*Theileria equi*-infected erythrocytes were incubated with trtE or DMSO vehicle control and proliferation was measured after 72 hours by SYBR green incorporation. Percent proliferation was calculated relative to DMSO treated controls. Inhibition of proliferation was analyzed as described for (**A**), EC_50_=391 pM (95% CI 286-550 pM).
