# Supplementary material for: A symbiotic bacterium of shipworms produces a compound with broad spectrum anti-apicomplexan activity
Source: PLoS Pathog. 2020 May 26;16(5):e1008600. doi: 10.1371/journal.ppat.1008600 (PMC7274485; doi:10.1371/journal.ppat.1008600)
Supplement: S12 Fig — B. bovis CE11/p2xHA-glmS-gfp-bsd parasites were treated with DMSO (top panel) or 50 nM trtE (bottom panel) for 24h prior to fixation and immunostaining. The infected erythrocytes were labeled with rabbit anti-GFP detected with goat anti-rabbit IgG (H&L chains)-Alexafluor 488 (green) to visualize the parasite cytoplasm and an anti B. bovis RAP-1 mouse mAb (MBOC79B1) detected with goat anti-mouse IgG (H&L chains)-Alexafluor 594 (red). Nuclei were counterstained with DAPI (blue). Left panels show the merger of the three color channels, middle panels show the fluorescence image overlaid the phase-contrast image and the right panels show the phase contrast image. Control panels show an early invaded erythrocyte (A) and mature meront (B), whereas the trtE-treated parasites (C and D) shown are divided meronts. Scale bars = 5 μm. (DOCX) [file ppat.1008600.s012.docx]

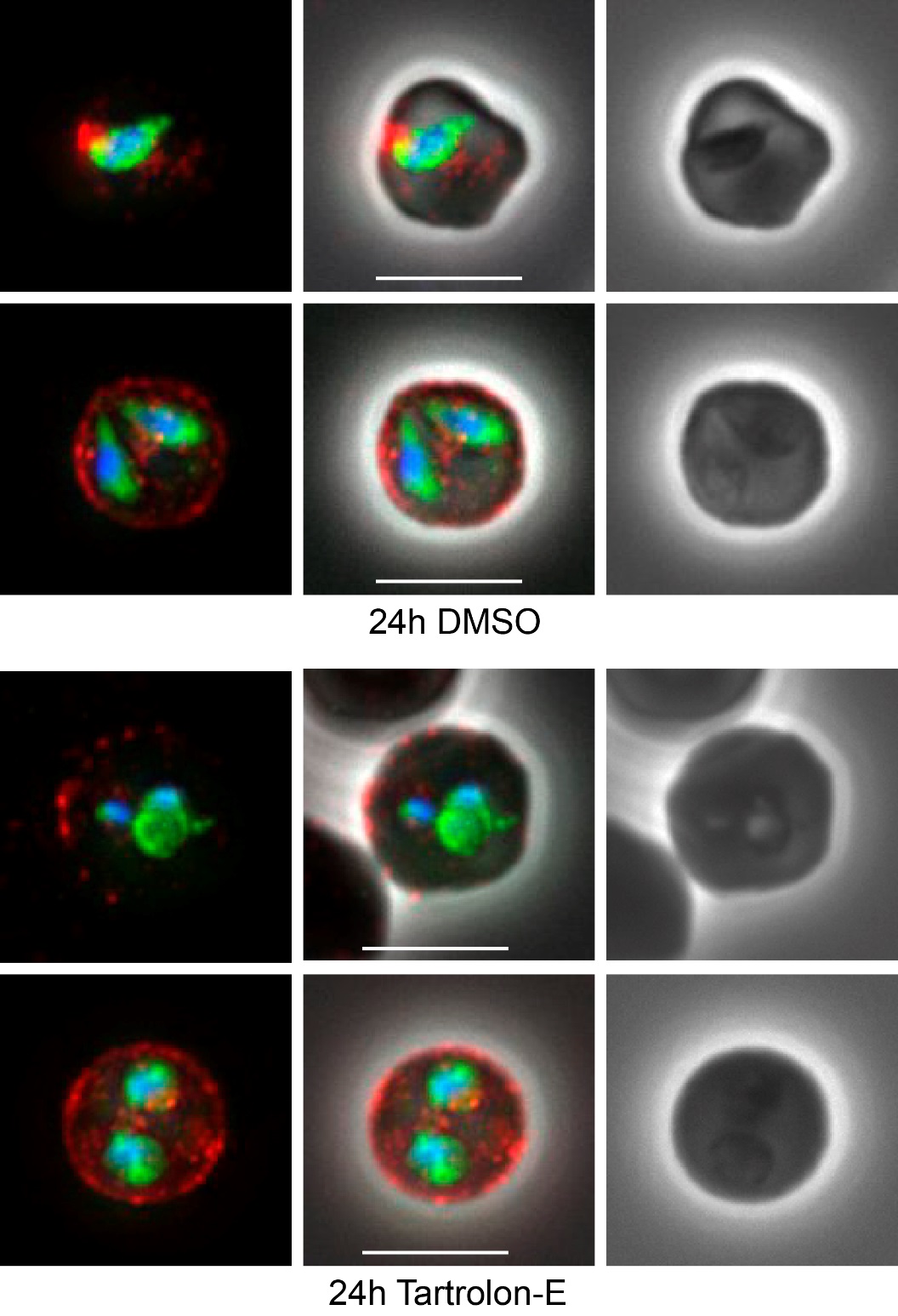


A

B

C

D

**S12 Fig: *Babesia bovis*-infected erythrocytes treated with trtE**: *B. bovis* CE11/p2xHA-glmS-gfp-bsd parasites were treated with DMSO (top panel) or 50 nM trtE (bottom panel) for 24h prior to fixation and immunostaining. The infected erythrocytes were labeled with rabbit anti-GFP detected with goat anti-rabbit IgG (H&L chains)-Alexafluor 488 (green) to visualize the parasite cytoplasm and an anti-*B. bovis* RAP-1 mouse mAb (MBOC79B1) detected with goat anti-mouse IgG(H&L chains)-Alexafluor 594 (red). Nuclei were counter-stained with DAPI (blue). Left panels show the merger of the three color channels, middle panels show the fluorescence image overlaid the phase-contrast image and the right panels show the phase contrast image. Control panels show an early invaded erythrocyte (A) and mature meront (B), whereas the trtE-treated parasites (C and D) shown are divided meronts. Scale bars = 5 μm.
