# Supplementary material for: A symbiotic bacterium of shipworms produces a compound with broad spectrum anti-apicomplexan activity
Source: PLoS Pathog. 2020 May 26;16(5):e1008600. doi: 10.1371/journal.ppat.1008600 (PMC7274485; doi:10.1371/journal.ppat.1008600)
Supplement: S13 Fig — Host cells were seeded into the wells of a 96 well plate to achieve approximately 25% confluency. 24 hours after seeding, trtE was added at the concentrations indicated. DMSO was run in parallel. 24 hours post addition of compound, viability of the cells was determined by quantification of ATP with CellTiter Glo. TC50s were determined using the log[inhibitor]vs response-Variable slope (four parameter) regression equation in Graphpad Prism. A. HFF: TC50 7.9 μM (95% CI 6.1–10.4), B. HCT-8: TC50 6.3 μM (95% CI 5–8), C. BT cells: TC50 16.8 μM (95% CI 11.6–28.8). (DOCX) [file ppat.1008600.s013.docx]

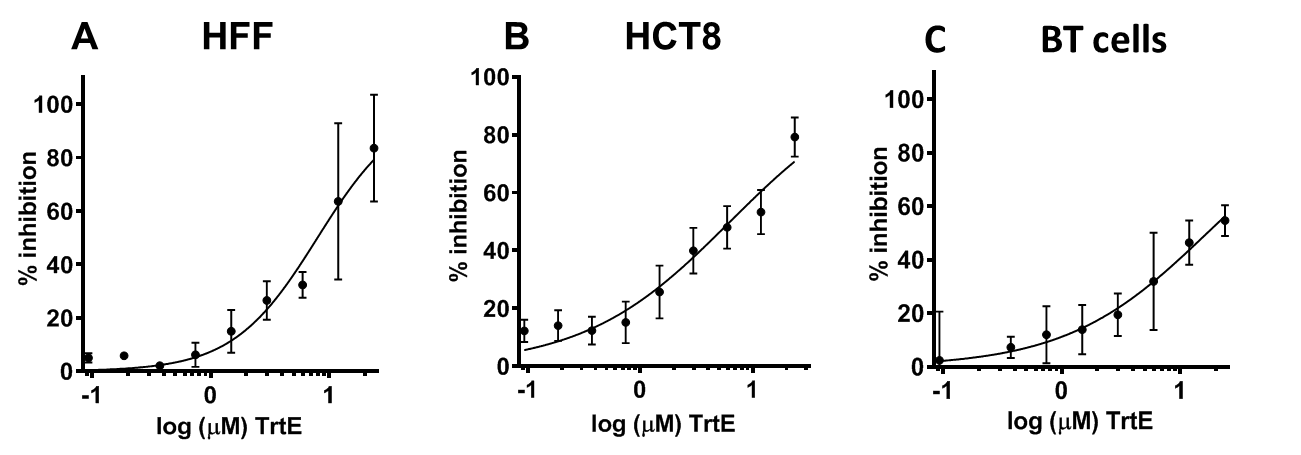


**S13 Fig: Cytotoxicity of trtE for host cells**. Host cells were seeded into the wells of a 96 well plate to achieve approximately 25% confluency. 24 hours after seeding, trtE was added at the concentrations indicated. DMSO was run in parallel. 24 hours post addition of compound, viability of the cells was determined by quantification of ATP with CellTiter Glo. TC_50_s were determined using the log[inhibitor]vs response-Variable slope (four parameter) regression equation in Graphpad Prism. **A**. HFF: TC_50_ 7.9 µM (95% CI 6.1-10.4), **B.** HCT-8: TC_50_ 6.3 µM (95% CI 5-8), **C.** BT cells: TC_50_ 16.8 µM (95% CI 11.6-28.8)
