# Supplementary material for: A symbiotic bacterium of shipworms produces a compound with broad spectrum anti-apicomplexan activity
Source: PLoS Pathog. 2020 May 26;16(5):e1008600. doi: 10.1371/journal.ppat.1008600 (PMC7274485; doi:10.1371/journal.ppat.1008600)
Supplement: Attachment — Submitted filename: Response to editors and reviewers.docx [file ppat.1008600.s016.docx]

Top of Form

Bottom of Form

| Date: | April 15, 2020 |
| --- | --- |
| To: | "PLOS Pathogens" plospathogens@plos.org |
| From: | Roberta O’Connor, rob.oconnor@wsu.edu |
| Subject: | PLOS Pathogens: Decision on PPATHOGENS-D-20-00071 |

Dear Drs. Striepen, Carruthers, Haldar and Malim,

We are resubmitting our manuscript “A symbiotic bacterium of shipworms produces a compound with broad spectrum anti-apicomplexan activity” revised in response to the editors and reviewer’s requests. We thank the editors and reviewers for their comments and we believe that our manuscript is significantly improved.

The major changes to the manuscript are:

1. An extensive re-write of the results section and the figure legends, and improved labeling of the figures to improve clarity of the manuscript.
2. Inclusion of DIC images in Figures 1B, 2B and 3B, and addition of Supplemental figures S1, S7, S10 and S12 to provide more images of trtE-treated parasites.
3. Additional discussion of specific topics requested by the reviewers.

Below are our specific responses (**in bold**) to the editors’ and reviewers’ comments (*in italics*). Line numbers refer back to the file named “TrtE manuscript revised”. All changes to the manuscript are tracked in this file.

We hope that the editors and reviewers now will find the manuscript much improved and worthy of publication.

Regards,

R.M. O’Connor

Veterinary Microbiology and Pathology

Washington State University

Pullman, WA, 99163

rob.oconnor@wsu.edu

**Editors comments:**
 *1. Please word your statements carefully. Fig. 1 e.g. claims “… supernatants exhibited parasiticidal activity that inhibited the growth of T. gondii”. So did they kill or just inhibit growth? In this experiments the authors only measure growth and thus should qualify their statement accordingly. If they stand by kill as a core to their claim, that can be tested in T. gondii using drug wash-out, lysis and plaque assay. Please comb the entire manuscript carefully to match claim with strength of data.*

**We have carefully reviewed the document and edited any statements that were not entirely accurate. For example in lines 132-133, “killed” is changed to “inhibited growth”, and line 277, “TrtE kills parasites” was changed to “TrtE kills *T. gondii* parasites”.**

*2. The reviewers had several question about how growth was quantified in particular for Cryptosporidium. One reviewer suggested the use of luciferase parasites, which the authors actually did. Part of the issue here may be the inconsistent labeling of the figures throughout which confused the reviewer and may well do so for readers, it distracted the editor. This is likely due to the fact that different co-authors prepared different panels, but “editorial cleanup” from the lead author could really help the clarity. Please settle on uniform fonts, sizes and colors. One reviewer suggested to clearly indicate the parasite species in each panel in a consistent fashion -- that is an excellent suggestion. Also take a careful look at the legends and make sure they give full detail. This is a relatively brief article, so there is room for clear description.*

**We have extensively re-written the results section and figure legends to improve clarity and to provide consistent language throughout. We have corrected the labeling and the fonts so that they are the same in all figures-those included in the manuscript and the supplemental figures as well. We have indicated the parasite species in Fig 3, and in Fig 2 we have included the stage of the parasite used in the assay in the y-axis legends. In the supplemental figures, where appropriate, we have labeled the individual graphs with the name of the parasite or host cell.**

*3. The reviewers have a number of important questions about toxicity and other aspects that may weigh in on the future use of these compounds including synthesis and isolation and pharmacokinetics. Please discuss these thoroughly, clarify your data as requested and supply further information on toxicity. Potential users of a cryptosporidiosis drug in particular (there is treatment for toxoplasmosis) will require a high safety margin, this is particularly true for pediatric applications.*

**We have included a discussion of these topics in the manuscript (lines 343-359) and we have added a sentence noting the lack of overt toxicity in trtE treated neonatal mice (lines 272-273).**

*4. Some reviewers were unable to evaluate the supplementary movie files. The editor notes that they were in gif format which can be viewed with web browser but is not the typical format standard used. Please confer with editorial office for guidance (most use mov or avi). Also a running clock or time stamp would help to appreciate the result of this experiment.*

**We have included a timestamp in the movies and have converted the movies to .mp4 format as requested by PLOS Pathogens.**

***********************

**Reviewer's Critiques**

**Part II – Major Issues: Key Experiments Required for Acceptance**

*Reviewer #1: 1) Immunofluorescent images are not a good morphological tool, especially when the authors are showing only one image with no DIC or phase. While it would be better to have EM, at the very least Figures 1b, 2b, 3b and 3e must have the DIC or phase controls, multiple images taken and added to supplemental data, and the slides need to be blinded before pictures are taken to reduce bias.*

**We have now included the DIC images for 1b, 2b and 3b, and we have included more images of the IFAs in supplemental figures 1, 7 and 10. The phase contrast images for figure 3e are shown in supplemental figure 11. Our approach to blinding samples has been to encode the treatments, so that the person reading the slide and taking the pictures is blinded to treatment. However, the effects of trtE treatment on parasite morphology were so consistent and dramatic, it was always patently clear which sample received the drug.** **We have added details of how the samples were blinded in the methods (lines 448-449)**. **Please note that we had to repeat the assays with *Cryptosporidium* to obtain additional publication quality images (shown in Fig 3B and Suppl. Fig S10), and these assays were done using HCT-8s as the host cell. This has been noted in the methods (lines 450, 453-455)**

*2) For the extracellular Toxoplasma time course experiment (Fig 2e), it is unclear if no drug extracellular controls were done for the same time periods because Toxoplasma becomes rapidly unhealthy during extracellular incubations.*

**Yes, exactly, *Toxoplasma* does not survive very well under extracellular conditions. To control for this, we ran a DMSO control in parallel, and the control values were set to 100%, and all treated samples compared to the control. To improve clarity, we added more details to the description of the methods for this assay (line 509-510, and lines 512-514).**

*3) No direct evidence of Cryptosporidium growth arrest in tissue culture, as the immunofluorescence is not high quality. Isn’t there a luciferase containing Cryptosporidium strain? Why was that not used so that the tissue culture data could be quantitative. Otherwise, could the slides be blinded and the authors measure vacuole size, or could they do the qPCR?*

**We did quantify parasite inhibition by trtE using the nano-luciferase expressing *C. parvum* (figure 3A). As noted by the editors, the reviewer likely missed this experiment because of a lack of clarity in the figure legend. We have edited the figure legend (lines 855-863) and figure labeling to improve clarity.**

Reviewer #2: None

Toxicity to animals needs to be more thoroughly evaluated, as this is the major issue in developing ionophores into therapeutics.

**Complete evaluation of toxicity in in vitro assays and in animals are among our planned experiments once we have scaled up production of the compound. Although ionophores have been generally viewed as toxic, recent reports from the cancer field have found that some ionophores, salinomycin in particular, are efficatious against stem-cell like cells at doses far lower that the toxic dose. It is also important to note that ionophores are routinely included in poultry and ruminant feed to inhibit coccidiosis, and toxic levels are quite different between species. We have included a section in the discussion that elaborates on this topic (lines 343-359). We also note that in neonatal mice, even at doses of up to 40 mg/kg, no overt toxicity was observed (lines 272-273).**

To support TrtE as a "broad-spectrum" anti-apicomplexan compounds, in vivo activity against at least one more parasite may be needed.

**We agree and in vivo experiments against other parasites are planned. We have specified that trtE is broad spectrum against apicomplexan parasites *in vitro* (for example see lines 192-193).**

In mouse model cryptosporidial infection model, what were the oocyst counts in feces?

**Quantification of oocyst shedding is not possible in this particular model of *Cryptosporidium* infection as the dam is coprophagic and consumes the feces as she cleans the neonates. Hence, reduction in parasitemia is evaluated by histology and qPCR of the intestine. Despite this shortcoming, the neonatal mouse model has proven to be a robust predictor of drug efficacy against cryptosporidiosis in calves, as well as a feasible in vivo model in situations where compound is available in limited quantities.**

**Part III – Minor Issues: Editorial and Data Presentation Modifications**
Please use this section for editorial suggestions as well as relatively minor modifications of existing data that would enhance clarity.

**Reviewer #1**

*1) The results need more methods added so the reader can follow the experiments. For example, lines 149-156 are very confusing and it would help to add detail such as grown for 24 hours, then add drug for 24 hours.*

**We have edited the results throughout to add more method details and we have rewritten the problematic section to improve clarity (lines 153-169)**

*2) The movies in supplemental were not functioning.*

**We have changed the movies to .mov format and added time stamps**

*3) Figure 3a, c and d should be labeled with the parasite name so the reader can more easily follow this complicated figure.*

**This has been done for all the figures now**

**Reviewer #2***:*

*1) Did you collect plasma or GI tissues during mouse therapy and try to measure TrtE levels? As a point of reference, it would be nice to know if there was systemic exposure of TrtE. This could help guide route of administration for other systemic parasitic infections. It could help gauge what levels of systemic exposure occurs during Cryptosporidium therapy and compare that to toxicology measurements. Do you know anything about pharmacokinetics, in general, of TrtE. Or, if none of this is done/known, perhaps discussing this as something that should be done*.

**Unfortunately, we did not collect plasma or GI tissues from the mouse experiment for the purpose of quantifying trtE as at the time our resources were extremely limited. Since then, we have begun, a pilot pharmacokinetic study in lambs that suggests that trtE is rapidly absorbed from the intestine and is tolerated by the animals. A full PK/PD study of trtE in mice and lambs has recently been funded, so in the near future we will be able to answer these questions. As the reviewer suggests we have added to the discussion a paragraph discussing the experiments yet to be done that are necessary before trtE can be considered a viable drug candidate including compound production, toxicity and pharmacokinetics (lines 343-365).**

*2) Can you tell the reader how reproducibly this natural product is produced by this bacterial strain and in what quantities? There are two descriptions of two batches; has more been made or attempted?*

**Dr. Schmidt’s laboratory has done over 100 individual fermentations of *T. turnerae* T7901 over the course of the past 10 years. In all cases so far attempted, trtE is the major compound isolated from the cell pellets. Our average measured yield is currently ~10 mg/L. His lab has, in total, made and purified about 1.5 g over the years. The Clardy lab modified the purification method (method #2) but the yield per liter was the same. We have included this information in the discussion (lines 343-347).**

3) Can you tell the reader how difficult it appears to make sufficient quantities of TrtE reproducibly, so it could be used practically and commercially? Of if not, what steps are

**The reproducibility of trtE production is excellent. What is required for commercial production is strain improvement, for which there are many methods. Probably ~1 g / L would be commercially viable, especially given that the compound is sub-nanomolar for many applications. We have included this information in the discussion (lines 347-348).**

*Has any whole animal toxicity testing been done with TrtE and how much of a safety index is there compared with dosages needed for effective Cryptosporidium therapy. Or, if not done, perhaps discussing this as something that should be done.*

**Toxicity studies have not yet been done, but we have added a discussion of the need for toxicity studies to the manuscript (348-359). We also note that we did not observe overt toxicity in the neonatal mice treated with trtE (lines 272-273).**

*5) Do you know anything about TrtE activity against Toxoplasma gondii bradyzoites?*

**A very preliminary study indicated that trtE was not effective against bradyzoites except at concentrations close to the level of toxicity for the host cells (~1 µM), so we have not pursued this line of investigation.**

***Reviewer #3:***

*1. For the compound tartrolon E (trtE), it may be necessary to balancedly discuss some chemical features of trtE and analogs, and their known bioactivities.*

**We did discuss briefly the bioactivities of this class of compounds but we have added some more details on the chemical features of the tartrolons and related compounds (lines 302-309).**

*2. For rationale to explore a shipworm symbiotic bacterium as a source of new antiparasitic compounds, the introduction and some other parts of the manuscript was too extensive and unnecessary. Some statements were not well justified, such as the implication that shipworm symbiont needed to produce antibiotics to inhibit gregarine parasites, as gregarines usually do not cause too much harm to their hosts such as mollusks (at least having not been defined). It could be simply competing with other bacteria for local niche colonization.*

**TrtE has been reported to have anti-bacterial activity but the EC50 of trtE for bacteria is 10 to 100 fold higher that the EC50s of trtE for apicomplexans. It may indeed function in bacteria-bacteria interactions, but then why would the compound have evolved to be far more potent against apicomplexans? All the apicomplexans seem to be exquisitely sensitive to this compound, and we seek to explain the existence of such a compound in this symbiosis. While we agree (and we state) that the hypothesis that trtE may have evolved to prevent gregarine infection of shipworms is entirely speculative (lines 295-298), it is not unreasonable. While it is true that most gregarines (that we know about) appear to be non-pathogenic for their hosts, there are several instances where they are pathogenic (listed in a recent paper by Rueckert et al 2019[****1****]). It is also possible that this compound has broader activity than we are aware of, and may be effective against protozoans in general, and perhaps evolved as a defense against protozoan pathogens of shipworms. Alternatively, it is possible that the compound evolved to protect the free-living stages of *Teredinibacter* from predation against protozoans. (Although then why would the bacteria continue to expend energy to produce this compound while safe within the shipworm host? Unless perhaps trtE prevents protozoa from establishing in the shipworm gut.)**

**However, we agree with the reviewer we probably stated our theory too strongly and we have softened our language (see abstract, line 36 and introduction, lines 98-106) and removed a line from the author summary (lines 58-61).**

*3. The concept of Cryptosporidium as a gregarine was wrong. Cryptosporidium is not a gregarine, although they both are branched at the base of the Apicomplexa. Cryptosporidium and gregarines are also highly divergent in metabolism.*

**We refer to *Cryptosporidium* as a gregarine because the most recent literature on apicomplexan phylogeny includes *Cryptosporidium* in a new order, Cryptrogregarinorida, included in the subclass Gregarinasina (Adl et al, 2019[****2****]). This classification will no doubt be refined as more information is obtained about the gregarines of which very little is known and only a tiny fraction of which have been in any way characterized.**

**It is true that a genome sequence comparison between *Ascogregarina* and *Cryptosporidium* unearthed great differences in their metabolic capacity (Templeton et al 2010 [****3****]), but because of the tremendous diversity amongst the gregarines, a complete understanding of this subclass, and *Cryptosporidium’*s position within it, awaits broader more comprehensive comparisons.**

*4. For broad-spectrum of activity: In principle, all ionophores (structurally, tartrolons are ion-carriers) share the same mechanism of action for their bioactivity, i.e., disrupting ion balances across cell membranes by insertion into bio-membranes. Because their primary targets are not specific proteins or other molecules (as they act on bio-membranes), they typically have narrow margins of safety (as demonstrated by several anti-coccidial ionophore drugs). The authors may want to discuss the significance of the findings more balancely by highlighting these and related points.*

**We have added a paragraph to the discussion on potential toxicity of this compound (lines 348-359). It is not actually clear that trtE exerts it’s anti-parasitic effect via its K+ ionophore activity and we discuss these possibilities briefly (lines 332-342). It may interest the reviewer to know that we did attempt to demonstrate that trtE’s anti-*T. gondii* activity was via it’s potential K+ ionophore activity. Boromycin [****4****] and tartrolon B[****5****] have been shown to be K+ ionophores, and boromycin’s activity against Mycobacterium tuberculosis [****4****] can be ameliorated by the addition of excess KCl. We tried this experiment but the addition of excess K+ ions had no effect on trtE’s activity against extracellular tachyzoites. We are still looking for a positive control for this experiment, and for this reason did not include this data in the manuscript.**

*5. Figure 3: Please mark parasite names in individual figures for easy reading (e.g., in Y-axis or appropriate position).*

**Thank you for this suggestion! We did this in figure 3 as well as some of the Supplemental figures.**

**References:**

1. Rueckert S, Betts EL, Tsaousis AD (2019) The Symbiotic Spectrum: Where Do the Gregarines Fit? Trends Parasitol 35: 687-694.

2. Adl SM, Bass D, Lane CE, Lukes J, Schoch CL, et al. (2019) Revisions to the Classification, Nomenclature, and Diversity of Eukaryotes. J Eukaryot Microbiol 66: 4-119.

3. Templeton TJ, Enomoto S, Chen WJ, Huang CG, Lancto CA, et al. (2010) A genome-sequence survey for Ascogregarina taiwanensis supports evolutionary affiliation but metabolic diversity between a Gregarine and Cryptosporidium. Mol Biol Evol 27: 235-248.

4. Moreira W, Aziz DB, Dick T (2016) Boromycin Kills Mycobacterial Persisters without Detectable Resistance. Front Microbiol 7: 199.

5. Surup F, Chauhan D, Niggemann J, Bartok E, Herrmann J, et al. (2018) Activation of the NLRP3 Inflammasome by Hyaboron, a New Asymmetric Boron-Containing Macrodiolide from the Myxobacterium Hyalangium minutum. ACS Chem Biol 13: 2981-2988.
