# Supplementary material for: A symbiotic bacterium of shipworms produces a compound with broad spectrum anti-apicomplexan activity
Source: PLoS Pathog. 2020 May 26;16(5):e1008600. doi: 10.1371/journal.ppat.1008600 (PMC7274485; doi:10.1371/journal.ppat.1008600)
Supplement: Attachment — Submitted filename: Response to editors and reviewers#2.docx [file ppat.1008600.s017.docx]

Top of Form

Bottom of Form

| Date: | April 29, 2020 |
| --- | --- |
| To: | "PLOS Pathogens" plospathogens@plos.org |
| From: | Roberta O’Connor, rob.oconnor@wsu.edu |
| Subject: | PLOS Pathogens: Decision on PPATHOGENS-D-20-00071 |

Dear Drs. Striepen, Carruthers, Haldar and Malim,

We have revised our manuscript “A symbiotic bacterium of shipworms produces a compound with broad spectrum anti-apicomplexan activity” in response to the editors’ and reviewers’ requests. I have also recirculated the final version again to all the authors, and some made minor corrections to this final version. These corrections can be seen in the manuscript with the tracked changes.

Many thanks for the thoughtful and thorough review of our manuscript.

Regards,

R.M. O’Connor

Veterinary Microbiology and Pathology

Washington State University

Pullman, WA, 99163

rob.oconnor@wsu.edu

Specific responses to the critiques in **bold** below (line numbers given refer to the document containing the tracked changes):

line 216: cut 'another' as it is unclear whether coccidian refers to Toxoplasma or Cryptosporidium here ( a view no longer held by most in the field), just say the coccidian Sarcocystis **Done: line 209**

line 255: Some reviewers dearly hated the idea to call Cryptosporidium a gregarine but the authors call it gastrointestinal gregarine parasite in this headline. I suggest to call it a gastrointestinal parasite. Yes they are closer to gragarine than e.g. Toxoplasma is, but overall the phylogeny around that question is still somewhat debated. The editor has little interest in that debate and it's really not important to this paper. Plasmodium is not called a gregarine here -- but still killed by the drug. The authors have an interesting evolutionary model and they should be able to discuss that. They did, so let's keep the rest focused on what is well established, and that is that C. parvum is a gastrointestinal parasite. (BTW many gregarines are gastrointestinal parasites as well, just not of mammals). **In a couple of places we removed the word “gregarine” and replaced it with “gastrointestinal”. Lines 64 and 248**

line 276: were/was **Corrected line 269**

line 313 structural details **Added, line 306**

300-316: so this paragraph is really confusing in that it concludes that "tatralons are quite variable" and then "tartralons are very similar to each other". Please work this over to a clean and consistent claim. **We removed one sentence (Lines 307-308) and we believe this paragraph is now clear**
